# Posttraumatic stress symptoms in children diagnosed with type 1 diabetes

**DOI:** 10.1186/1824-7288-38-13

**Published:** 2012-04-26

**Authors:** Şahika G Şişmanlar, Emine Demirbaş-Çakir, Işık Karakaya, Filiz Çizmecioğlu, Cavit I Yavuz, Şükrü Hatun, Belma Ağaoğlu

**Affiliations:** 1 Department of Child and Adolescent Psychiatry, Kocaeli University Faculty of Medicine, Kocaeli, Turkey; 2 Department of Paediatrics, Section of Paediatric Endocrinology, Kocaeli University Faculty of Medicine, Kocaeli, Turkey; 3 Department of Public Health, Kocaeli University Faculty of Medicine, Kocaeli, Turkey; 4 Cumhuriyet mah. Ada sok. Türköz Sit. B-Blok Daire:3, Plajyolu-İzmit, Kocaeli, Turkey 41100

**Keywords:** Posttraumatic stress, Diabetes, Hypoglycaemia, Children

## Abstract

**Background:**

Studies consistently found remarkable rates of posttraumatic stress symptoms (PTSS) in children with chronic diseases. But, only one study had searched PTSS in children with diabetes, until now. So, the present study aimed to examine incidence rate and predictors of PTSS in children with type 1 diabetes.

**Method:**

PTSS were evaluated by Child Posttraumatic Stress Reaction Index in fifty four children with diabetes (aged between 8–18 years). This assessment was based on hypoglycaemia as the potential traumatic event. Children were also introduced a brief questionnaire about demographic and disease related information. Some other information was obtained from families, medical stuff and records. Among 54 children, forty two had complete information. Hence, to evaluate possible predictive factors related with PTSS, multiple regression analysis was conducted for 42 children.

**Results:**

18.5% of children were reported PTSS at severe or very severe level, and 51.9% were reported PTSS at moderate level or above. Multiple regression analyses were shown that child PTSS were not significantly related with possible predictive factors other than number of hypoglycaemic attacks for the last month.

**Conclusion:**

The study results support that posttraumatic stress symptoms are not rarely seen in paediatric patients with diabetes, and even if not severe, hypoglycaemic attacks may be perceived as traumatic by the children with diabetes. But, because of some limitations, the results should be carefully interpreted.

## Background

In Diagnostic and Statistical Manual of Mental Disorders, Fourth Edition, Text Revision, trauma is described as the experience of intense fear, helplessness, horror, or disorganized and agitated behaviour in response to exposure to an event -directly or as a witness- that caused or threatened serious injury or violation of body integrity [1]. And, trauma literature in children generally deals with the impact of traumatic events such as disaster (earthquake, flood…), war, sexual or physical abuse, domestic or community violence. But invasive medical procedures, serious and chronic illnesses can also cause posttraumatic stress reactions [2].

Studies of paediatric patients and/or their parents consistently found remarkable prevalence rates of posttraumatic stress symptoms (PTSS) and posttraumatic stress disorder (PTSD) for variety of chronic physical illnesses. For example, prevalence of PTSS and/or PTSD in paediatric patients with cancer varies across the studies and has been estimated between 2-20% in survivors, even many years after the end of cancer treatment [3,4]. Also, PTSS were found to be significantly related with asthma severity or life-threatening asthma in children [5,6]. In a study with inner city children with severe asthma, 25% of the sample met the criteria for PTSD [5].

Given that type 1 diabetes mellitus (Type 1 DM) is a life-threatening, chronic illness, it could be a precipitant of PTSD for patients and their family members. But, reviewing studies on relationship between diabetes and trauma/PTSD, adult studies are predominantly seen. And, two kinds of link were addressed about the relationship between diabetes and trauma thereby PTSD. First, researchers has suggested that responses to traumatic stressors appear to have a physiological foundation that could result in a host of human diseases [7,8]. For example, exposure to traumatic life events (e.g. childhood abuse) may lead to increased likelihood of physical illnesses such as diabetes [9,10]. And, impact of PTSD on physical health was also mentioned in studies with war veterans [11] although some were not supported this association [7], for review [12]. On the other hand, posttraumatic stress reaction may be developed secondary to diabetes, associated with a life-threatening episode. This is consistent with the finding of Myers (2003) who reported high rates of PTSD in adult patients with type 1 DM [13]. Beside the studies with adult patients, the studies with parents of children with diabetes have also stressed increased prevalence of PTSS or PTSD in parents after diagnosis of a chronic illness, diabetes for their children [14].

There is only one study in English literature, examining PTSS and/or PTSD in paediatric age group with type 1 DM. In this study, Landolt et al. examined rates and predictors of PTSD and PTSS in a large sample of paediatric patients with cancer, diabetes and history of accidents, and compared the results of children with the results of their parents. Children with newly diagnosed type 1 DM showed lowest rates of PTSS, the incidence of moderate-severe posttraumatic stress reaction was 5.4%. In general, reaction index (RI) scores of children were found to be significantly associated with their functional status [15].

As the relationship between PTSS and diabetes has been a rarely studied subject in paediatric age group, this study is aimed to examine rate of PTSS and predictive factors that could be related with PTSS in a group of paediatric patients with type 1 DM.

## Methods

### Participants and procedure

The study group was consisted of 54 children, aged between 8–18 years with type 1 DM. They were recruited among children who had been attending to “Diabetes Summer Camp” coordinated by Paediatrics Department of Kocaeli University Medical School at 1–8 August 2010. The camp is organized yearly and takes 7 days. Some aims of the camp are to inform children about diabetes, to establish and to strengthen awareness about the disease, to help them in improving coping strategies about physical and psychosocial problems that they face with during their daily life because of a chronic disease, diabetes. Expert team of the camp included one child and adolescent psychiatrist, 1 psychologist, 1 child development expert, 7 paediatricians, 4 student doctors, 4 nurses, and 2 dieticians, 2 student dieticians, 2 swimming teachers, and 2 students of physical education and sports working on recreation. Additionally, there were 9 experienced adolescents with diabetes who were older than the children in the camp, so being a model for them. Before the summer camp, informed consents were obtained from all of the parents about all the medical procedures and mental evaluations. During the camp period, education was given to the children about diabetes, daily. After the education program, Child Posttraumatic Stress Reaction Index (CPTS-RI) and a brief demographic questionnaire were introduced to all the children at the camp (n = 79). 54 of the children at the camp were voluntary for this evaluation and filled CPTS-RI and the questionnaire. Moreover, some information about functional level, glucose control and disease related parameters were obtained from paediatricians, nurses and also from medical records. Functional level was assessed with regard to physical activities, functioning at school and interpersonal relationship. And, glucose control denoted the patient’s ability to balance their blood glucose level, obtained by diabetic team. Information about hypoglycaemic attacks for not only the camp period but also for the last month had been gathered from the blood glucose charts of children noted at home and patients’ files. Blood glucose level ≤60 mg/dl was accepted as hypoglycaemia and presence of one of the followings during a hypoglycaemic attack: blood glucose level ≤30 mg/dl, loss of consciousness, requirement of glucagon injection or parenteral treatment at hospital was the criterion for severe hypoglycaemia. Among 54 children, the information resources other than child himself were not able to be reached for 12 children. So, 42 children were completed the study.

### Measures

#### Demographic questionnaire

A brief questionnaire about demographics and disease related information was introduced to participants. This information included age, gender, educational level of children and their parents, family situation, socioeconomic status, diabetes duration, frequency of hypoglycaemic attacks (number of attacks for the last month), number of diabetic ketoacidosis, traumatic life events (natural disaster, combat experience, life-threatening accident, witnessing someone badly injured or killed, rape, sexual molestation, physical attack or assault, threatened with a weapon or held captive, tortured, or the victim of terrorists). The information on demographic questionnaire was also checked with parents and diabetic team.

#### Child posttraumatic stress reaction index (CPTS-RI)

Child Posttraumatic Stress Reaction Index is designed to assess reactions following various traumatic events in school-age children and adolescents. It is a self-reported, 5-point Likert scale ranging from “none of the time” (0) to “most of the time” (4) and contains 20 items. A total score is obtained by summing of all items after adjusting for reverse-scored items. A total score of 12–24 indicates a mild level of PTSD; a score of 25–39 indicates a moderate level; a score of 40–59 indicates a severe level and; a score above 60 indicates a very severe level of reaction to the trauma. First and second items of the scale explore Category A (presence of a traumatic life event) of DSM diagnostic criteria for PTSD; the items 3, 4, 5, 6, 17, 19 are for the assessment of Category B (intrusion); the items 7, 8, 9, 10, 14, 16 are for Category C (avoidance) and the items 11, 12, 15, 20 are for Category D (arousal). The remained 2 items are related with grief and regression. In the present study, subscale scores for each symptom category of PTSD were calculated by summing the scores of each items related to that symptom category.

The RI had been developed by Pynoos et al. [16]. Translation into Turkish and validity and reliability study for Turkish children were done, and it demonstrated a good test-retest reliability (0.86) and interrater reliability (.98) [17].

### Statistical analysis

Statistical analyses were performed with the SPSS 16.0 statistical package. For demographics and CPTS-RI scores, categorical measures (e.g. gender, family situation) were compared using Chi-square tests. Mann–Whitney U tests were used for the continuous variables. Correlational analyses were carried out by Pearson Correlation analyses.

To identify variables that effect CPTS-RI total score or PTSS level, multiple regression analysis was conducted. The statistical significance level was taken as p ≤ 0.05.

## Results

Mean age of 54 children who had filled CPTS-RI was calculated as 13.67 with a standard deviation of 2.39. Table1 summarizes CPTS-RI mean scores of 54 children. 51.9% of these children reported PTSS at moderate level or above, and 18.5% were reported PTSS at severe-very severe level. Comparing children with complete data (n = 42) and with incomplete data (n = 12), there was no significant difference in age, gender, age and educational level of parents, average per capita income, and CPTS-RI mean scores (Table1). Then, analysis was carried on for children with complete data. Demographical and disease related parameters were not significantly different between children with above and below severe-very severe level of PTSS (Table2).

**Table 1:** Patient characteristics and mean values of CPTS-RI scores

	**Total group (n = 54)**	**Complete data group (n = 42)**	**Incomplete data group (n = 12)**	**Statistical Analyses**
Age in years, mean ± SD	13.67 ± 2.39	13.67 ± 2.37	13.67 ± 2.61	Z = −0.063, p = 0.950*
Female (%)	27 (%50)	21 (%50)	6(%50)	*X*^2^ = 0.000, p = 1.000**
Age of mother, mean ± SD	39.91 ± 6.10	39.62 ± 6.45	40.92 ± 4.76	Z = −0.917, p = 0.359*
Education of mother, mean ± SD	6.50 ± 3.06	6.50 ± 3.01	6.50 ± 3.37	Z = −0.070, p = 0.944*
Age of father, mean ± SD	45.15 ± 6.90	44.64 ± 6.97	46.92 ± 6.65	Z = −1.053, p = 0.292*
Education of father, mean ± SD	8.80 ± 3.56	8.60 ± 3.39	9.50 ± 4.17	Z = −0.573, p = 0.567*
Average per capita income, mean ± SD	364.02 ± 242.26	370.18 ± 253.92	342.45 ± 204.47	Z = −0.375, p = 0.707*
**CPTS-RI***Mean score ± SD*				
Intrusion	8.54 ± 5.94	8.83 ± 5.50	7.50 ± 7.44	Z = −1.148, p = 0.251*
Avoidance	7.65 ± 4.97	7.76 ± 4.97	7.25 ± 5.19	Z = −0.417, p = 0.677*
Hyper arousal	4.67 ± 2.93	4.79 ± 2.99	4.25 ± 2.77	Z = −0.157, p = 0.875*
Total Score	26.37 ± 1.39	26.71 ± 1.26	25.17 ± 1.87	Z = −0.614, p = 0.539*
*% with total score*				
No PTSS	8 (%14.8)	3 (%25)	5 (%11.8)	*X*^2^ = 7.795, p = 0.099**
Mild PTSS	18 (%33.3)	5 (%41.7)	13 (%31.0)	
Moderate PTSS	18 (%33.3)	1 (%8.3)	17 (%40.5)	
Severe PTSS	9 (%16.7)	2 (%16.7)	7 (%16.7)	
Very severe PTSS	1 (%1.9)	1 (%8.3)	0 (%0)	

*Mann–Whitney *U* Test, **Chi-square Test.

**Table 2:** Characteristics of children with diabetes

**N = 42**	**CPTS-RI < 40 (n = 35)**	**CPTS-RI ≥ 40 (n = 7)**	**Statistical Analysis**
Age in years, mean ± SD	13.46 ± 2.39	14.71 ± 2.06	Z = −1.279, p = 0.201*
Female (%)	17 (%48.6)	4 (%57.1)	*X*^2^ = 0.171, p = 0.679**
Lives with both biological parents (%)	31 (%88.6)	6 (%85.7)	*X*^2^ = 0.895, p = 0.639**
Age of mother, mean ± SD	38.69 ± 5.42	44.29 ± 9.36	Z = −1.184, p = 0.237*
Education of mother, mean ± SD	6.66 ± 3.13	5.71 ± 2.36	Z = −1.204, p = 0.229*
Age of father, mean ± SD	44.34 ± 6.58	46.14 ± 9.15	Z = −0.118, p = 0.906*
Education of father, mean ± SD	8.83 ± 3.45	7.43 ± 3.05	Z = −1.078, p = 0.281*
Average per capita income (New Turkish Lira), mean ± SD	381.06 ± 269.49	315.83 ± 157.87	Z = −0.186, p = 0.852*
Stressful life events, mean ± SD	0.34 ± 0.59	0.29 ± 0.49	Z = −0.086, p = 0.932*
Family history of diabetes (%)	17 (%48.6)	4 (%57.1)	*X*^2^ = 0.171, p = 0.679**
Family history of other chronic diseases (%)	14 (%40.0)	2 (%28.6)	*X*^2^ = 0.323, p = 0.570**
Family history of mental disorders (%)	3 (%8.6)	0 (%0)	*X*^2^ = 0.646, p = 0.421**
Duration of diabetes, mean ± SD	3.49 ± 2.75	5.14 ± 4.67	Z = −0.818, p = 0.414*
HbA_1c_ (DCCT unit, %), mean ± SD	8.09 ± 1.86	6.97 ± 1.10	Z = −1.486, p = 0.137*
HbA_1c_ (IFCC unit, mmol/mol), mean ± SD	65 ± 2.03	53 ± 1.21	
Number of diabetic ketoacidosis, mean ± SD	0.46 ± 1.09	0.29 ± 0.49	Z = −1.181, p = 0.856*
Hypoglycaemic attacks for the last month, mean ± SD	7.11 ± 6.89	13.57 ± 15.34	Z = −0.678, p = 0.498*
Severe hypogycemia (%)	9 (%25.7)	3 (%42.9)	*X*^2^ = 0.840, p = 0.359**
Control of blood glucose level (%)			
Good	13 (%37.1)	3(%42.9)	*X*^2^ = 1.188, p = 0.552**
Moderate	12 (%34.3)	1(%14.2)	
Bad	10 (%28.6)	3(42.9%)	
Functional status (%)			
Good	15(%42.9)	4(%57.1)	*X*^2^ = 0.599, p = 0.741**
Moderate	9(%25.7)	1(%14.3)	
Bad	11(%31.4)	2(%28.6)	

*Mann–Whitney *U* Test, **Chi-square Test.

As possible predictive variables for development of PTSS, all of the demographical, disease related parameters, CPTS-RI mean scores and PTSS severity rates were entered to Pearson Correlation analysis. We determined no significant correlation (p ≤ 0.05).

In order to construct models to predict development of PTSS in children with diabetes, we used multiple regression analyses. Three predictor variables were included in each model, based on study hypothesis and correlational analyses. Many models were tried, and predictor variables, entered to the analysis in general were age, gender, age and education of parents, family history of diabetes and other chronic diseases, number of hypoglycaemic attacks fort he last month, history of any severe hypoglycaemia or ketoacidosis, HbA_1c_ level, functional and glucose control level of children with diabetes. Table3 summarizes the results of regression analysis. Among variables, only “number of hypoglycaemia for the last month” was significant predictor of PTSS in children with diabetes (Table3).

**Table 3:** Regression analysis

	**ß**	**p**
**Constant**	24.410	0.043
**Hypoglycaemic attacks per month**	**0.450**	**0.041**
**Age**	−0.169	0.845
**Familial history of diabetes**	9.680	0.164

*Durbin-Watson value = 1.736.

## Discussion

In this study, prevalence rate and predictive factors for development of PTSS were tried to be explored in children with type 1 DM. In the group of 54 children, 18.5% showed severe-very severe level of PTSS. As possible predictive factors for PTSS, age, gender, socioeconomic level, properties of parents, family structure, family history of diabetes and other chronic diseases, traumatic life events, number of hypoglycaemia, history of any severe hypoglycaemia or ketoacidosis, HbA_1c_ level were evaluated. But, other than “number of hypoglycaemia for the last month”, no relationship was found between PTSS and these potential predictive factors.

There are data suggesting high rates of PTSD (25.5%) in adult patients with type 1 DM [13]. However, PTSD or PTSS in children with diabetes have been studied only in one study to date. Landolt et al. (2003) found that 5.4% of paediatric patients with diabetes expressed at least moderate level of PTSS [15]. On the other hand in our study, 18.5% of children had severe-very severe level of PTSS, and 51.9% of them had moderate level of PTSS or above. Comparing two studies, it is thought that discrepancy in prevalence rates of PTSS might be due to methodological differences. In the study of Landolt et al., the study group consisted of children with newly diagnosed diabetes, and the children were assessed 5–6 week after the diagnosis [15]. But in our study, duration of diabetes changed between 1–12 years. One more difference between two studies is about mean age of the children. Children aged between 6.5–14.5 years (mean age 10.4 ± 2.5) were recruited for the study of Landolt et al. [15]. Children were between 8–18 years of age, and mean age was calculated as 13.67 ± 2.39 in our study group. Although no relationship between age or disease duration and PTSS could be shown in our analyses, the discrepancy between two studies might be the result of that children in our study group were older, and have been living with diabetes for longer period, they might have been experienced more negative life events and might raise more awareness for their disease, diabetes. PTSD prevalence of 25.2% in adults with type 1 diabetes in the study of Myers [13] also supports this assumption.

Many possible predictive factors for the development of PTSS in children with diabetes were put in to analyses. Besides the factors that had been evaluated in the previous study such as age, gender, socioeconomic status, traumatic life events and functional level [15], some other factors such as family status, familial history of chronic diseases, number of hypoglycaemic episodes, presence of any severe hypoglycaemia and ketoacidosis, control of blood glucose were also handled. But, other than number of hypoglycaemic attacks per month, none of the factors were found to be associated with PTSS development.

In some studies, assessment of PTSD or PTSS in patients with chronic diseases have been carried out upon the rationale that receiving a diagnosis of chronic disease may be sufficient to qualify as traumatic event [1,18]. However in our study, PTSS assessment was based on hypoglycaemia as the potential traumatic event, like in the study of Myers [13]. Myers (2003) is the first researcher who examined the impact of hypoglycaemic episodes related to diabetes as a potential source of PTSD on adult patients [13]. Our study was also based on the knowledge that fear of hypoglycaemia often results in increased and chronic anxiety among many individuals with DM. But, PTSS was found related with frequency of hypoglycaemic episodes but not with experience of severe hypoglycaemia. Moreover, our study group did not present any link between PTSS and functional level which had been a predictive factor in the study of Landolt et al. [15]. We should not forget that our study group is a selected one. Apart from their family, they were at a camp program which included educational programs about diabetes, and potential dangers arising out of diabetes. Moreover, during camp period, they might witness to hypoglycaemic experiences of other children. So, although they didn’t experience severe hypoglycaemia, due to increased awareness about dangers and witnessing, hypoglycaemia itself may trigger PTSS in camp children, thereby high PTSS rates of the study group. And, more than a severe hypoglycaemic attack experienced any time in the past, milder but new attacks might be more effective on PTSS development. Additionally, due to presence of only few numbers of children with a history of severe hypoglycaemia, we may not statistically find any relation with PTSS.

The results of high PTSS rate in children with diabetes, relation of PTSS with frequency of hypoglycaemia but not with severity, and with other potential predictive factors may be due to small sample size. Secondly, physical or psychological symptoms experienced during a hypoglycaemic attack may lead the child to misinterpret them as PTSS. So, assessment of hypoglycaemic attacks in depth may provide more proper evaluation for further researches. Thirdly, although we tried to assess many possible risk factors for PTSS, some other important traumatic life events secondary to diabetes (social exclusion, rejection or bullying by the peers, not expressing about her disease, stigmatization or accusation by peers or officials because of injector usage) was out of our assessment. As non of the predictive factors other than number of hypoglycaemia was associated with PTSS, a biological vulnerability for PTSS in patients with type 1 DM should be a question kept in mind.

Some limitations of the present study should be addressed. Small sample size and studying with a selected group limit generalization of the study results. Additionally, not evaluating children in depth about hypoglycaemic attacks or hypoglycaemic fear, and inadequate assessment of possible predictive factors for PTSS development in children with diabetes are the other associated limitations of our study.

## Conclusion

The study results support that posttraumatic stress symptoms are not rare in paediatric patients with diabetes, and even if it is not severe, a hypoglycaemic attack may be perceived as traumatic by the child with diabetes. So, paediatricians and other health workers should be sensitive and careful for PTSS in children with diabetes. Comparing number of studies about PTSD in children with other chronic diseases, studies on PTSD in children with diabetes are very few in number, most of which dealt with PTSD or PTSS in parents of the patients, and only one searched PTSS in children with type 1 DM. It shows that traumatic life events especially hypoglycaemia and development of PTSS in children with diabetes have been ignored both in research area and in clinical practice. Therefore, further studies on larger sample groups, containing in dept evaluation of hypoglycaemic attacks together with other possible traumatic life events are needed in the future.

## Abbreviations

PTSS: Posttraumatic stress symptoms; PTSD: Posttraumatic stress disorder; Type 1 DM: Type 1 diabetes mellitus; CPTS-RI: Child Posttraumatic Stress Reaction Index; HbA_1c_: Glycated haemoglobin.

## Competing interest

The authors declare that they have no competing interests.

## Authors’ contributions

ŞGŞ and IK designed the study. ŞGŞ, IK and EDÇ conducted the electronic research and initial screening. EDÇ extracted the data. CIY conducted the data analysis. ŞGŞ and IK wrote the first draft and subsequent revisions. IK and FÇ reviewed the article. ŞGŞ, IK, FÇ, EDÇ and CIY contributed the interpretation of results. BA and ŞH provided the expert advice. All authors read and approved the final manuscript.

## Author details

^1^Department of Child and Adolescent Psychiatry, Kocaeli University Faculty of Medicine, Kocaeli, Turkey. ^2^Department of Paediatrics, Section of Paediatric Endocrinology, Kocaeli University Faculty of Medicine, Kocaeli, Turkey.^3^Department of Public Health, Kocaeli University Faculty of Medicine, Kocaeli, Turkey. ^4^Cumhuriyet mah. Ada sok. Türköz Sit. B-Blok Daire:3, Plajyolu-Izmit, Kocaeli, Turkey41100.
